# Transcriptional time-course analysis during ash dieback infection revealed different responses in tolerant and susceptible *Fraxinus excelsior* genotypes

**DOI:** 10.1186/s12870-025-06074-z

**Published:** 2025-01-25

**Authors:** Víctor Chano, Renata Callegari Ferrari, Tania Domínguez-Flores, Karuna Shrestha, Barbara Fussi, Hannes Seidel, Oliver Gailing, Katharina B. Budde

**Affiliations:** 1 Department of Forest Genetics and Forest Tree Breeding, University of Göttingen, Büsgenweg 2, Göttingen, 37077 Germany; 2 Center for Integrated Breeding Research (CiBreed), University of Göttingen, Albrecht-Thaer-Weg 3, Göttingen, 37075 Germany; 3 University of Göttingen, Carl-Sprengel-Weg 1, Göttingen, 37075 Germany; 4 Forestry Development Department, Oak ParkCarlow, R93 XE12 Ireland; 5 Bavarian Office for Forest Genetics (AWG), Forstamtsplatz 1, Teisendorf, 83317 Germany; 6 Northwest German Forest Research Institute, Professor-Oelkers-Straße 6, Hann. Münden, 34346 Germany

**Keywords:** European ash, *Hymenoscyphus fraxineus*, RNA-Seq, Time-series, Gene expression analysis

## Abstract

*Hymenoscyphus fraxineus*, the causal agent of Ash Dieback (ADB), has been introduced to eastern Europe in the 1990s from where it spread causing decline in European ash populations. However, the genetic basis of the molecular response in tolerant and susceptible ash trees to this disease is still largely unknown. We performed RNA-sequencing to study the transcriptomic response to the disease in four ash genotypes (ADB-tolerant FAR3 and FS36, and ADB-susceptible UW1 and UW2), during a time-course of 7, 14, 21, and 28 days post-inoculation, including mock-inoculated trees as control samples for each sampling time point. The analysis yielded 395 and 500 Differentially Expressed Genes (DEGs) along the response for ADB-tolerant FAR3 and FS36, respectively, while ADB-susceptible UW1 and UW2 revealed 194 and 571 DEGs, respectively, with most DEGs found exclusively in just one of the genotypes. DEGs shared between tolerant genotypes FAR3 and FS36, included genes involved in the production of phytoalexins and other secondary metabolites with roles in plant defense. Moreover, we identified an earlier expression of genes involved in both pattern- and effector-triggered immunity (PTI and ETI) in ADB-tolerant genotypes, while in ADB-susceptible genotypes both responses were delayed (late response). Overall, these results revealed different transcriptomic expression patterns not only between ADB-tolerant and ADB-susceptible genotypes, but also within these two groups. This hints to individual responses in the natural tolerance to ADB, possibly revealing diversified strategies across ash genotypes.

**Supplementary Information:**

The online version contains supplementary material available at 10.1186/s12870-025-06074-z.

## Introduction

In recent history, several outbreaks and pathogenic diseases have had an enormous impact on forests worldwide. For instance, chestnut blight caused by *Cryphonectria parasitica* was first reported at the beginning of the twentieth century, and it is still affecting American and European *Castanea* populations [60], and the two epidemics of the Dutch elm disease (DED) that killed about 90% of elms in natural and urban areas of Europe (*Ulmus minor* Mill. and *Ulmus laevis* Pall.) and North America (*Ulmus americana* L.; [54]). Moreover, tree diseases have also been causing significant harm in forest ecosystems in more recent years. One example is the ash dieback (ADB hereinafter), which is a fungal disease caused by the invasive pathogen *Hymenoscyphus fraxineus* that has been decimating European ash (*Fraxinus excelsior* L.) populations since the beginning of the 1990s, when it was first observed in Poland [64]. Since then, the disease has severely affected ash populations towards the north [82] and the west, reaching France in 2008 and Spain in 2021 [77]. In Germany, increased mortality due to ADB has been observed for the last 20 years [29]. The ascomycete *H. fraxineus* originates from Asia and did not co-evolve with *F. excelsior*. Nevertheless, numerous studies have shown that susceptibility of European ash to ADB varies and is a heritable trait (reviewed in [22]). The fungus spreads via airborne ascospores that infect leaves and petioles of *Fraxinus* trees [34]. When the pathogen grows through the petiole-shoot junction into woody tissue it causes necrosis and shoot dieback affecting the crown and causing cankers on branches and the stem [73]. Recurrent infections over several years weaken the host and are often lethal, with a mortality ranging between 70–85% [15]. Infected leaves are shed in autumn and the pathogen develops black pseudosclerotia on petioles and leaf rachises in the litter from which white fruiting bodies (apothecia) emerge and release the ascospores in summer of the following year [82].

To overcome diseases, plants rely on the innate immunity [7], which is based on i) the pattern-triggered immunity (PTI), and ii) the effector-triggered immunity (ETI). The defensive mechanisms of host plants start after the release of microbial- and pathogen-associated molecular patterns (MAMPs/PAMPs) subsequent to the attack of the pathogen. These molecules are detected by the hosts pattern-recognition receptors (PRRs), activating a cascade of responses that initiate the PTI (reviewed in [8]), and subsequent processes like the influx of Ca^2+^ ions from the extracellular space, the burst of reactive oxygen species (ROS), or the transcriptional reprogramming through the activity of mitogen-activated protein kinases (MAPKs). However, pathogens have developed the ability to promote the infection by interfering with the PTI by effector proteins, and plant hosts may in turn target pathogen effectors and initiate the ETI (reviewed in [18]. The ETI is considered as a second layer of plant immunity aiming to restrict the infection by means of a broader enhanced response and even by promoting programmed cell death (PCD, [7]). The ETI, or intracellular immunity, is initiated by nucleotide binding (NB) leucine-rich repeat (LRR) proteins (NLRs) through a model based on the gene-for-gene concept, where specialized receptors encoded by host resistance (R) genes target specific effectors encoded by the pathogen avirulence (Avr) genes [26].

In recent years, great efforts have been made to disentangle the molecular basis of resistance to ADB, with special focus on the identification of molecular markers that allow to discriminate between tolerant and susceptible individuals. For example, Harper et al. [36] and Sollars et al. [74] used associative transcriptomics to identify gene expression variants and SNPs associated to crown damage caused by ADB. One RNA-based marker, discovered by Harper et al. [36], revealed a moderate capacity to discriminate between tolerant and susceptible genotypes [56]. Using genome wide association analyses, SNPs significantly associated with disease symptoms were also identified and used to develop genomic prediction models for tree health with high accuracy [21, 55, 76]. Moreover, Chaudhary et al. [12] screened 63 amplicon derived candidate SNPs for their association with ADB-tolerance in Sweden and found only one significantly associated marker. These studies revealed a polygenic architecture of ADB susceptibility with many genes with small effects. However, confirmation of marker effects found in one study in other studies is limited (e.g., [78]). Recent ADB research has also incorporated transcriptomic studies to explore gene expression patterns at a single time point during the infection, highlighting differences between tolerant and susceptible individuals at a single time point [69],[25]). However, to our knowledge no transcriptomic study has thoroughly examined the immune response of ash trees against ADB or focused on the temporal development of the disease in leaves, even though time is an important factor in the development of this disease and the response might vary during the vegetative growth of the individuals [53].

In the current study, we performed a time-course analysis to advance in the understanding of early ADB resistance mechanisms by revealing the dynamic immune response of ash trees in leaves of susceptible and tolerant genotypes. The typical infection of *F. excelsior* with *H. fraxineus* occurs via the leaf surface. Therefore, leaf inoculations are needed to study the response of the host to the pathogen during an early stage of ADB. In a first leaf infection experiment [25], we conducted a gene expression analysis at 7 days post-inoculation (dpi hereinafter) in ash genotypes FAR3 and UW1, showing tolerance and susceptibility to ADB, respectively. This study revealed first transcriptional differences between the two genotypes and indicated that 7 dpi represents a rather early time point to study the transcriptional response to the infection. In the present study, we have extracted and sequenced RNA from 96 samples following a multifactorial time-series design, including four ash genotypes provided by the Bavarian Office of Forest Genetics (Bayrisches Amt für Waldgenetik, AWG): the already mentioned genotypes FAR3 and UW1, and additionally FS36 and UW2 showing tolerance and susceptibility to ADB, respectively. Moreover, samples were collected at four time points, 7, 14, 21, and 28 dpi, with three biological replicates for each treatment (inoculated and mock-inoculated) and genotype. Sequencing data was used to perform differential gene expression analysis, aiming at characterizing the temporal course of the ADB triggered transcriptomic response in European ash.

## Material and methods

### Plant material and experimental design

European ash trees were grown in a clonal common garden in Grabenstätt and monitored since 2014 by the Bavarian Agency of Forest Genetics (Bayrisches Amt für Waldgenetik, AWG). For this study, four genotypes were selected being identified as tolerant (FAR3 and FS36) and susceptible (UW1 and UW2) (Supplementary Figure S1; [71]). Twenty-four scions (ramets) from each of these genotypes were grafted in February 2021 onto rootstocks of two-year-old ash trees (provenance *F. excelsior* 81,101, north-west Germany, provided by Erwin Vogt Baumschulen GmbH, Pinneberg, Germany) and grown for one year in a greenhouse at the Department of Forest Genetics and Forest Tree Breeding, University of Göttingen. Trees were kept in 4 L plastic pots with commercially available substrate mixture (“Profi-Linie mineralisch” from Kleeschulte Erden GmbH & Co. KG, Rüthen, with pH (CaCl_2_) 6.0, Salinity 1.5 g/L, N 320 mg/L, P_2_O_5_ 120 mg/L, K_2_O 350 mg/L, Mg: 120 mg/L) and regularly watered. They were later repotted and fertilized with a universal plant fertilizer (Wuxal, Maag, concentration suggested by the manufacturer). The trees were exposed to biocontrol agents (*Amblyseius californicus* provided by Katz Biotech AG, Bayruth) against spider mites two times, before and during the experiment. In spring 2022, one month before inoculations, trees were transferred to climate chambers with a 16 h photoperiod (light intensity of 80 ± 15 mmol m^−2^ s^−1^, constant temperature of 19° ± 3 °C and relative humidity at 65 ± 10%) (Supplementary Figure S2a).

### Pathogen inoculation and sampling

Isolates of *H. fraxineus* used for inoculations were acquired and handled as described in Ferrari et al. [25]. Briefly, the culture collection at Julius Kühn Institute (JKI: Federal Research Centre for Cultivated Plants, Institute for Forest Protection, Braunschweig, Germany) representing a wide range of *H. fraxineus* strains induced symptom development in stems and petioles of ash trees [67]. The most virulent strain (Strain 7—RH03-T2-B1-1, deposited in the German Collection of Microorganisms and Cell Cultures—DSMZ, Braunschweig, Germany as DSM 116307) was used as inoculant in the present work.

The cultures of *H. fraxineus* were plated on MYP medium (prepared mixing 2.8 g malt, 0.4 g peptone, 0.2 g yeast, 6 g agar, 400 ml ultrapure water, and ca. 5 g ash leaves before autoclaving) and kept at room temperature for three weeks. Half of the plants were inoculated with round agar plugs (0.6 cm diameter, superficial sections) containing active mycelium by making an approximately one cm long superficial wound on one leaf petiole with a sterile scalpel (Supplementary Figure S2b). The other half of the plants were mock-inoculated with sterile MYP medium and used as control plants (Supplementary Figure S2c). In both groups, the parafilm which maintained the agar plugs in place was not removed until the moment of sampling. Petioles of three ramets (used as biological replicates) per genotype and treatment (inoculated and mock-inoculated) were sampled at each sampling time point (7, 14, 21, and 28 dpi), always starting at 10:00 am. At the time of sampling, petiole lengths of approximately 3 cm were cut, including the wound and peripheric regions (Supplementary Figure S2d-e), then placed in autoclaved 2 ml tubes, flash-frozen in liquid N_2_, and kept at -60 °C until use.

### RNA isolation, assessment of inoculation success and sequencing

Approximately 60 mg of petiole tissue was ground in a Retsch MM300 (F. Kurt Retsch, Haan, Germany) and RNA was extracted using the E.Z.N.A. Plant RNA Kit (Omega Bio Tek, Norcross, USA, R6827-01) according to the manufacture protocol for difficult samples. The concentration and purity of the RNA was assessed using a microvolume spectrophotometer (NanoDrop 2000, Thermo Fisher Scientific, Waltham, USA). Total RNA samples were treated with DNase I, RNase-free (Thermo Fisher Scientific).

These samples were used for the assessment of the inoculation success (data not shown). For this goal, cDNA synthesis was performed using 1 µg of RNA input and the SuperScript IV First-Strand Synthesis System (Thermo Fisher Scientific). Real-time quantitative polymerase chain reaction (RT-qPCR) was performed in an TOptical Gradient 96 Thermal Cycler (Biometra—Analitik Jena, Jena, Germany), using 10 µl reaction mix composed of 5 µl innuxMIX qPCR DSGreen Standard 2x (IST Innuscreen GmbH, Berlin, Germany), 2 µl cDNA sample (20 ng/µl) and 300 nM of forward and 300 nM of reverse primers. The amplification program consisted of a 2 min initial step at 95 °C, followed by 40 cycles with 10 s at 95 °C and 30 s at 60 °C, as suggested by the innuxMIX manual. In all cases, the melting curve was analyzed to detect unspecific amplification and primer dimerization. The fungal housekeeping gene *UBIQUITIN-CONJUGATING ENZYME E2* (*UBC*) [75] was used to assess the presence of the pathogen *H. fraxineus*. The primer sequences used for *UBC* were: forward primer 5' – CCTCGGACTCTCCATACTCG – 3'; reverse primer 5' – GATAGATTCTGGTGGTGAAGTT – 3'. Inoculations were considered successful when the number of threshold cycles (C_T_) < 30, while controls showed C_T_ > 30 or undetected (data not shown).

Only samples that passed the criteria for successful inoculation (C_T_ < 30) were used for RNA-sequencing together with uninfected control samples. The RNA quality (RQN) was assessed using a Fragment Analyzer System (PROSize 3.0, 3.0.1.5, 2015, Advanced Analytical Technologies, Agilent Technologies, Santa Clara, USA). cDNA libraries were obtained with the Stranded mRNA Prep kit (Illumina, San Diego, USA), which uses oligo(dT) magnetic beads for purifying and capturing polyA tails from mRNA molecules, and were sequenced using the NovaSeq 6000 platform, 100 bp and paired-end reads.

### Bioinformatic processing of sequencing data

A GNU/Linux based High-Performance Computing (HPC) system from the Gesellschaft für Wissenschaftliche Datenverarbeitung mbH Göttingen (GWDG) was used for the bioinformatic processing of sequencing raw data and downstream analysis. Initial quality control of sequencing reads (fastq files) was performed using FastQC v0.11.7 and MultiQC v.1.10.1 [23], and Trimmomatic v0.36 [10] was used to detect and remove Illumina adapter sequences together with 12 nucleotides from the head (HEADCROP routine) and 2 nucleotides from the tail (CROP routine) of the reads. Reads shorter than 20 nucleotides using a sliding window of size four were filtered out when the average phred score in the window was below 15. The new FRAX_001_PL version of the *F. excelsior* reference genome [55] (https://www.ncbi.nlm.nih.gov/datasets/genome/GCA_019097785.1/) was first indexed using the routine hisat2-build from Hisat2 v.2.1.0 [46], and the resulting pre-processed reads were then mapped with the option –score-min L,0,-0.2. After converting mapped sam files to bam format, reads were sorted by position in the genome and PCR duplicates were flagged with markdup using Samtools v.1.9 [49]. Final bam files were used as input for the software HTSeq v.2.0.2 [3] to create counting tables, using the annotation file (GFF3) of the FRAX_001_PL genome to indicate mRNA positions in the genome. One final table was obtained for each genotype, FAR3, FS36, UW1, and UW2, which were then imported to R v.4.2.0 [65] using the RStudio Server v.2022.06.0 from the GWDG home system.

### Differential gene expression analysis

The gene expression matrix generated for the four genotypes was imported in R for differential gene expression analysis using the R package DESeq2 v.1.36.0. [52]. The expression data was normalized by means of the variance stabilizing transformation (vst), and an exploratory Principal Component Analysis (PCA) of the transformed data was conducted. Subsequently, the differential gene expression analysis was performed following a Wald test (WT) to obtain logarithmic fold change (LFC) values between inoculated and mock-inoculated trees and a likelihood ratio test (LRT) to identify significantly differentially expressed genes (DEGs) across the series of time points (7, 14, 21, and 28 dpi) in a time-course analysis.

LFC values were obtained after the WT (indicated in DESeq by the formula design = ~ treatment) from the ratio between the expression values of inoculated and mock-inoculated trees. Therefore, LFC results in a positive value when a transcript was expressed higher in the infected samples (indicated as induced or up-regulated) or in a negative value otherwise (indicated as repressed or down-regulated). DEGs were considered significant when the p-value (adjusted for multiple comparisons by False Discovery Rate, FDR) was lower than 0.05, and LFC was greater than 2 in absolute values (*p*-value < 0.05; LFC >|2|). Sets of DEGs from each time point for each genotype were then compared using Venn diagrams by means of the R package vennDiagram [13], while the R package upsetR [16] was used to compare all the groups (time points and genotypes). Results from the WT were used to compare transcriptomic dynamics of the individual responses by time and genotypes, although no further interpretation of gene families, responsive networks and functionalities was considered.

For the time-course analysis, the LRT for each independent genotype was performed including the time (dpi) and the interaction of treatment and time as variables in the model formula (indicated as design = ~ treatment + time + treatment:time), while a reduced model (indicated as reduced = treatment + time) was used to identify genes with expression changes due to the treatment at any time point. For the LRT, DEGs were considered significant when adjusted (FDR) p-values were lower than 0.01 and LFC (previously obtained from WT) was greater than 2 in absolute values for at least one time point. The R package vennDiagram was then used to compare the set of DEGs from each genotype. A more stringent p-value threshold considered for the LRT, compared to the WT, allowed for the identification of the genes and pathways/networks with strong effect in the responses to ADB. Moreover, a hierarchical clustering of LRT-resulting DEGs from each of the four genotypes was performed using the Wards minimum variance method and based on Euclidean distances from LFC values obtained from WT. For the four genotypes, six clusters were defined from resulting dendrograms according to their expression profiles.

Functional annotation of genes was already described in Ferrari et al. [25], using 41,355 gene model sequences provided for the ash genome version FRAX_001_PL. In brief, a local protein database from RefSeq Viridiplantae (National Center of Biotechnology Information, NCBI) was used as reference for BLASTp using TOA v.0.66 [58], and Gene Onthology (GO) terms were later retrieved using Omicsbox v3.0.30 [32]. The full annotation dataset was then used as reference for a two-tailed simple enrichment analysis (SEA) of GO terms associated to the DEGs obtained from LRT for each genotype by means of Fishers exact test featured by Omicsbox v3.0.30 [1].

## Results

### Sequencing data

In this work, we performed RNA-seq of European ash leaves infected with *H. fraxineus*, the causal agent of ADB. Infected tissue was harvested from the inoculation area in the petiole at 7, 14, 21, and 28 dpi from two ADB-tolerant (FAR3 and FS36) and two ADB-susceptible ash genotypes (UW1 and UW2). In addition, a set of mock-inoculated trees of all genotypes served as control samples and were harvested at the same time points. In total, RNA-sequencing yielded 8,706 Mio 100 bp reads (45.34 Mio paired-end reads per sample). Stringent filtering was performed on raw sequencing data, resulting in 4,195 Mio high quality reads in total (43.70 Mio paired-end reads per sample). Mapping rates ranged from 89.14% to 99.25% per sample (average 96.88%).

### Differential gene expression at single time points: Wald Test

**Fig. 1:**
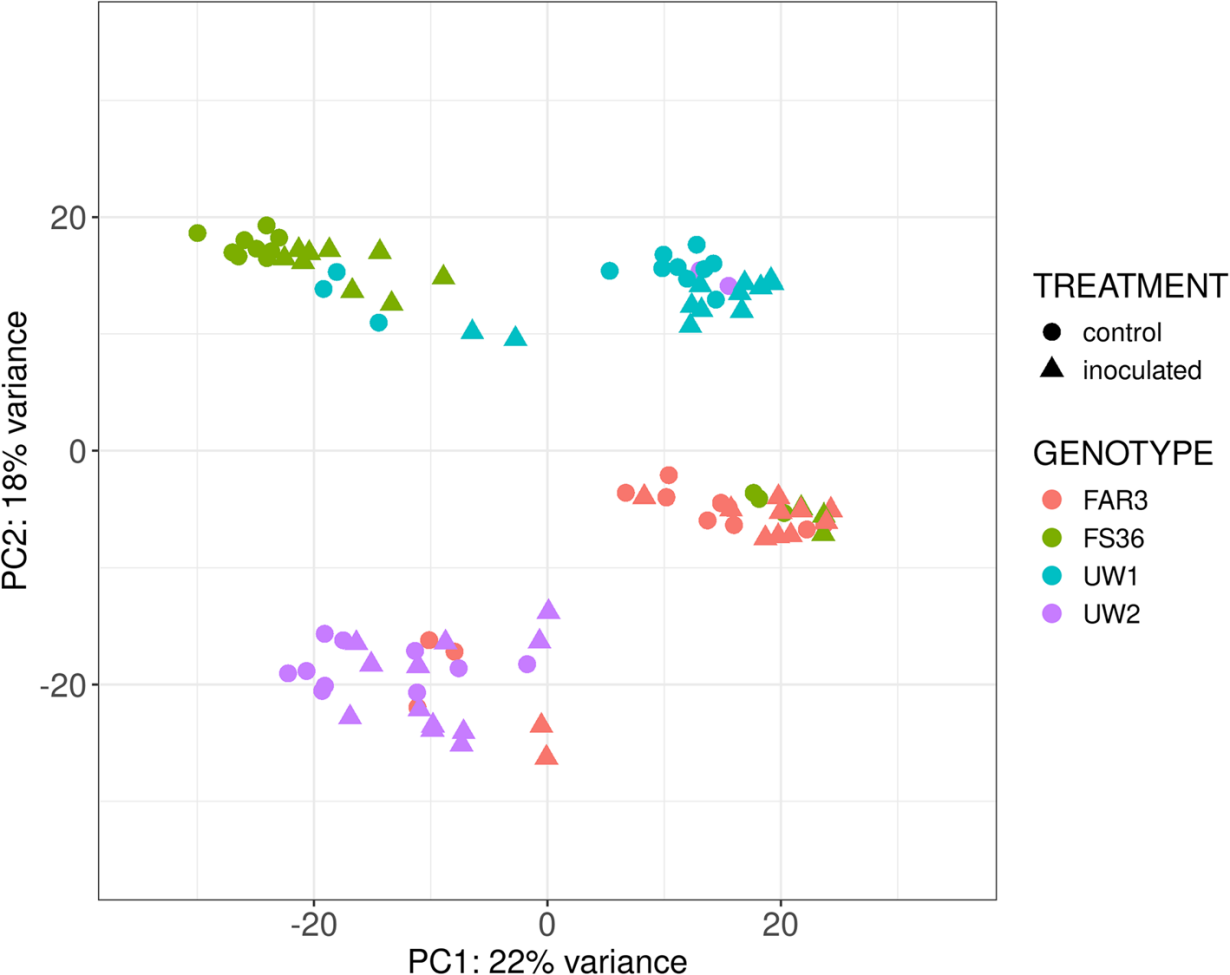
Principal component analysis (PCA) of transcripts sequenced in 96 *Fraxinus excelsior* samples, using normalized expression values by vst (variance stabilizing transformation), explaining 22% of the variance along the first component (PC1) and 18% of the variance along the second component (PC2)

For the WT analysis, each genotype and time point were tested independently by comparing both infected and mock-inoculated samples. The PCA of transformed expression values explained 22% and 18% of the variance along the first (PC1) and the second component (PC2), respectively (Fig. 1). With some exceptions, the four genotypes could be clearly separated by both PC1 and PC2, although the separation between treatments (inoculated and mock-inoculated) and time points was not always clearly distinguishable.

**Fig. 2:**
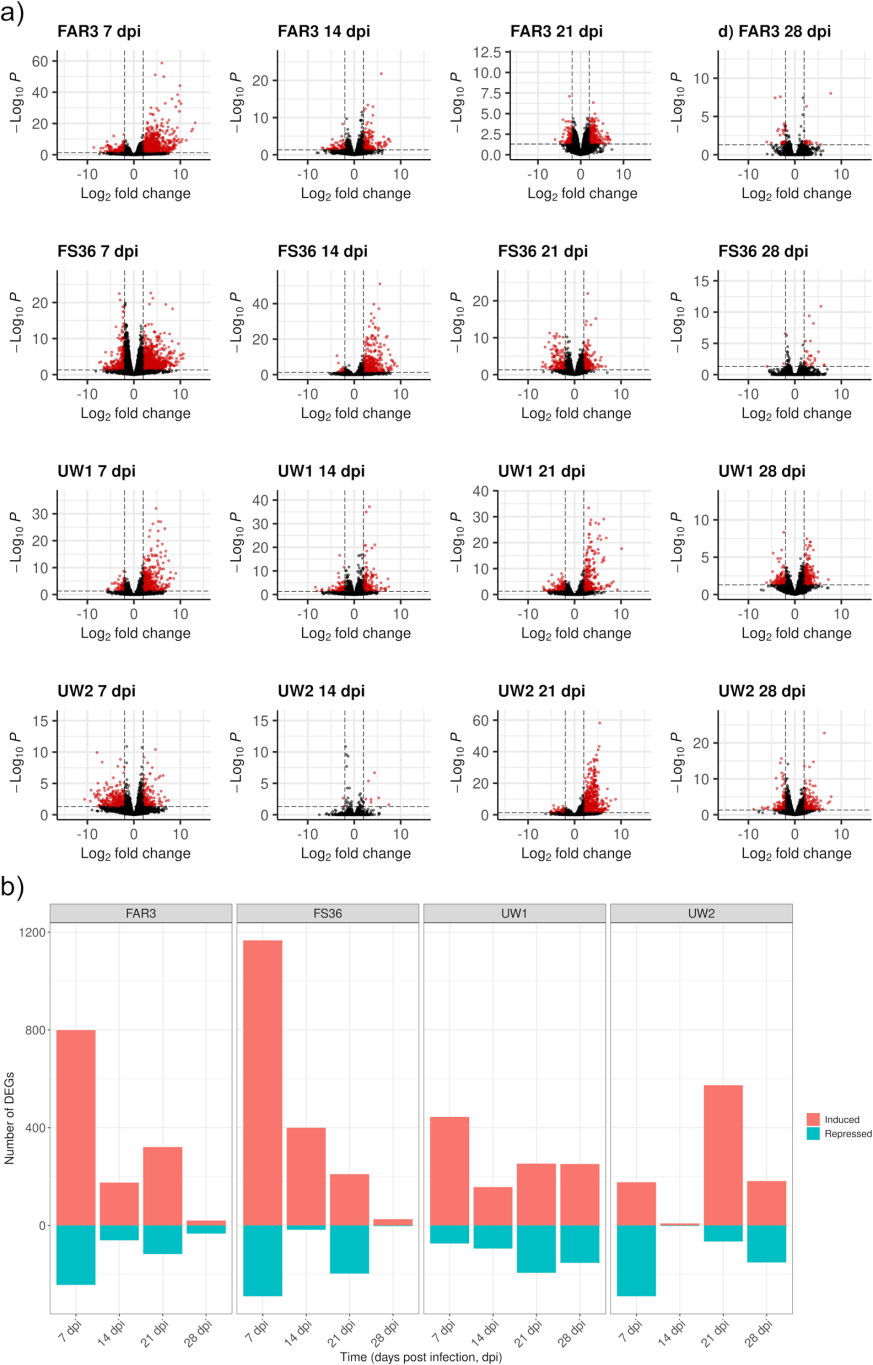
Genotype- and time-specific induced and repressed genes in inoculated versus mock-inoculated *Fraxinus excelsior* samples. **a** Volcano plots indicate differentially expressed genes (DEGs, red dots) between inoculated and mock-inoculated samples for each sampled time point (7-, 14-, 21- and 28-days post inoculation, dpi), and genotype (ADB-tolerant FAR3 and FS36, and ADB-susceptible UW1 and UW2). Dashed lines indicate thresholds for significance (*P* < 0.05, false discovery rate (FDR)-adjusted, horizontal) and expression values (Log_2_ fold change of |2|). **b** Barplots representing the number of DEGs for each time point and genotype. For visualization purposes, the numbers of induced genes are presented as positive values while the numbers of repressed genes are presented as negative values

In Fig. 2a, the overall distribution of DEGs from WT (adjusted *p*-value < 0.05, and LFC >|2|) is shown for each genotype and time point. At 7 dpi, most of the DEGs found in ADB-tolerant FAR3 and FS36 and ADB-susceptible UW1 were induced in inoculated plants, while the number of repressed DEGs in ADB-susceptible UW2 was higher. Moreover, the level of significance (including both induced and repressed DEGs) was higher in FAR3, according to the y-axes in the volcano plots. At 14 dpi, the number of induced genes was higher than repressed genes for the four genotypes, although UW2 showed an overall low number of DEGs compared to the other three genotypes. At 21 dpi, the distribution of induced and repressed DEGs for ADB-tolerant genotypes was more balanced, while ADB-susceptible genotypes showed a higher number of repressed transcripts, with a higher level of significance as shown along the y-axis. Finally, at 28 dpi, the number of DEGs for ADB-tolerant FAR3 and FS36 was lower compared to ADB-susceptible UW1 and UW2.

The numbers of significantly induced and repressed transcripts at the different time points and for the four genotypes are included in Supplementary Table S1, as well as in Fig. 2b, showing differences in the intensity of the transcriptional response when comparing induced versus repressed genes. On the one hand, and apart from smaller differences especially at 14 and 21 dpi, the transcriptional profiles of the two ADB-tolerant genotypes FAR3 and FS36 were similar, with a high transcriptional up-regulation at 7 dpi and with a pronounced drop in the response at 28 dpi. On the other hand, the two ADB-susceptible genotypes showed markedly different response levels, with a pronounced up-regulation in UW1 at 7 dpi, followed by a nearly stable level of response at 14, 21 and 28 dpi, but an unsteady level of response in UW2, highly down-regulated at 7 dpi, almost no significant response at 14 dpi, and followed by a sharp up-regulation at 21 dpi. Results from WT for each genotype and time point are shown in Supplementary Tables S2 to S17.

**Fig. 3:**
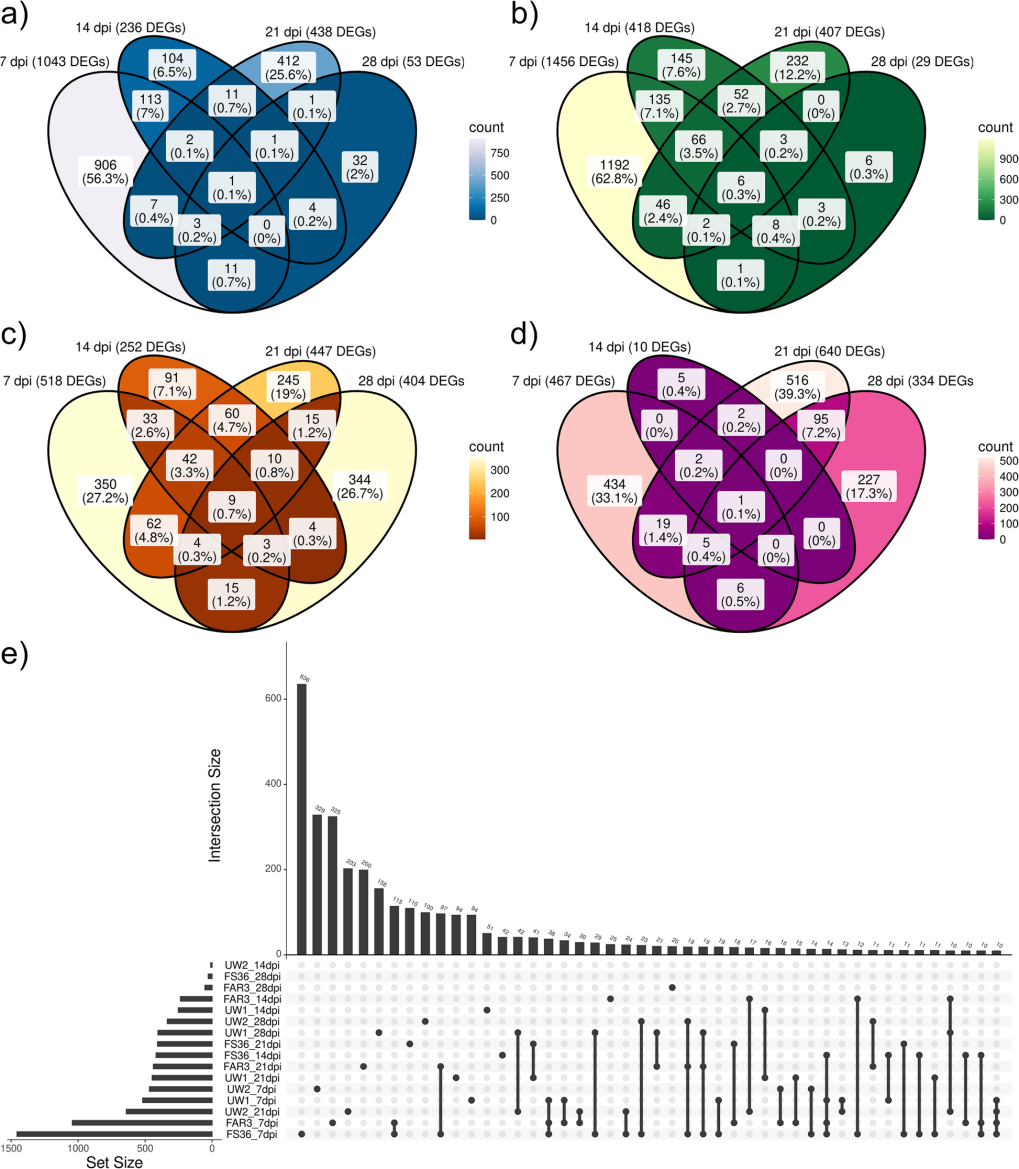
Shared and exclusive differentially expressed genes (DEGs) among genotypes and time-points in inoculated versus mock-inoculated *Fraxinus excelsior* samples. **a**-**d** Venn diagrams of differentially expressed genes (DEGs) from gene expression analyses following Wald test of each sampled time point (7-, 14-, 21- and 28-days post inoculation, dpi) for genotypes FAR3, FS36, UW1 and UW2, respectively. **e** Upset plot of DEGs resulting from the Wald test performed for each of the four time points and the four genotypes. The number of intersections is limited to a maximum of 10 DEGs

The number of exclusive DEGs for each sampling time point within genotypes was considerably higher than shared DEGs over time points. In FAR3, 906 DEGs (56.3%) were found to be exclusive at 7 dpi, while 412 (25.6%) were found exclusively at 21 dpi (Fig. 3a). A similar response was also found in FS36 (Fig. 3b), with 1192 exclusive DEGs (62.8%) at 7 dpi and 232 exclusive DEGs (12.2%) at 21 dpi. For the ADB-susceptible UW1 (Fig. 3c), the proportion of exclusive DEGs found at 7 dpi (350 genes, 27.2%) and 21 dpi (245 genes, 19%) was similar, but a large proportion of DEGs was also found to be exclusive at 28 dpi, with 344 genes (26.7%). Slightly different results were observed for UW2 (Fig. 3d), as 434 DEGs (33.1%) and 227 DEGs (17.3%) were exclusive at 7 and 28 dpi, but the larger group was found at 21 dpi, including 516 DEGs (39.3%). When comparing between time points and genotypes, the intersections also showed that the larger sets of DEGs were exclusive for individual genotypes at single time points (Fig. 3e; Supplementary Table S18). With 115 DEGs, the highest number of overlapping DEGs between the two ADB-tolerant genotypes was found at 7 dpi, in contrast to the ADB-susceptible genotypes at the same time point with only seven DEGs. The largest set of shared DEGs between the two ADB-susceptible genotypes, 42 DEGs, was found at different time points (28 dpi for UW1 and 21 dpi for UW2).

### Time-course analysis: likelihood ratio test

**Fig. 4:**
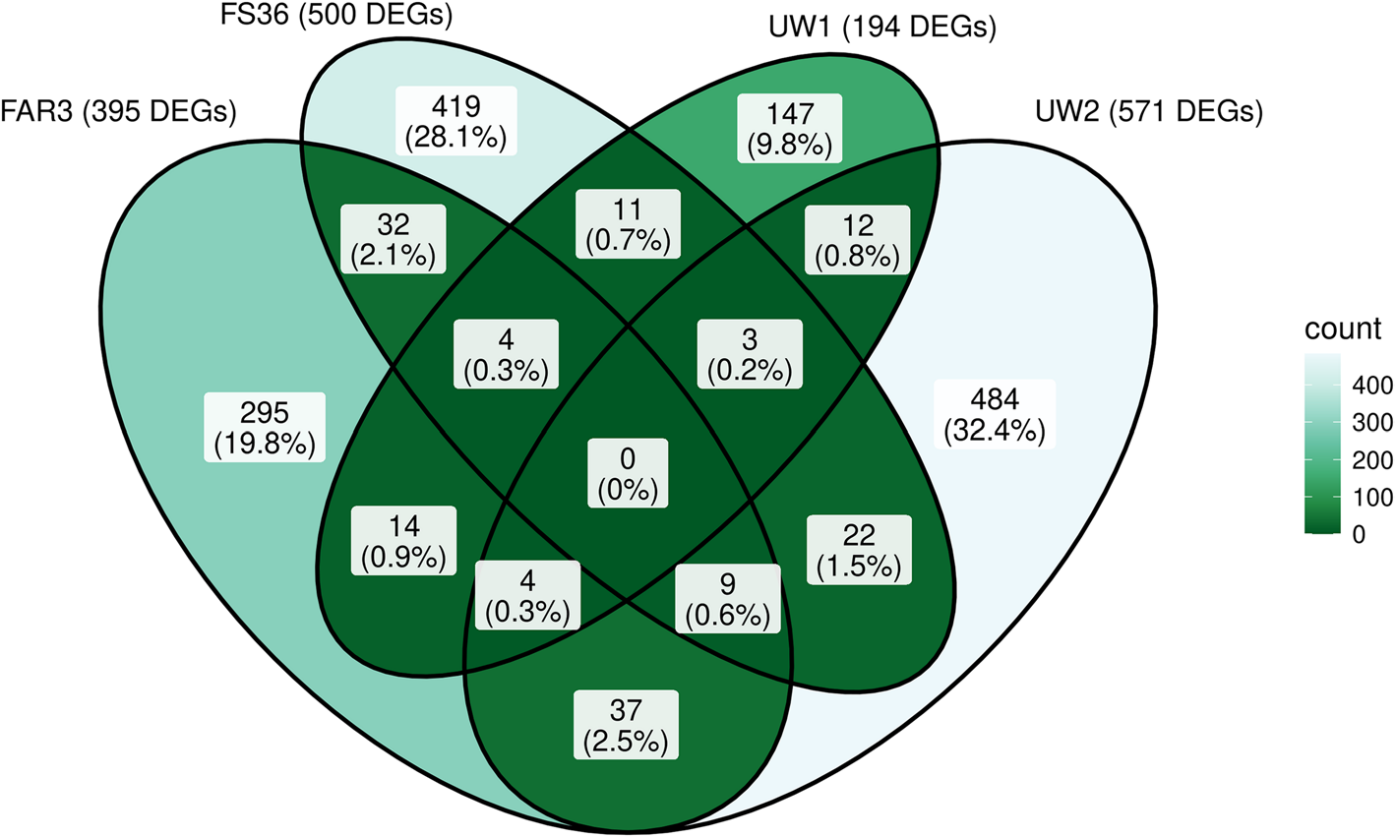
Venn diagram of differentially expressed genes (DEGs) from time-course gene expression analysis in inoculated versus mock-inoculated *Fraxinus excelsior* samples following the likelihood ratio test (LRT) for the four genotypes, ADB-tolerant FAR3 and FS36 and ADB-susceptible UW1 and UW2

The influence of time on the response to ADB of each genotype was analysed by performing a LRT and selecting as DEGs those transcripts showing adjusted p-values < 0.01 and LFC >|2| in at least one sampling time point (LFC values were derived from WT, where inoculated plants were compared to mock-inoculated plants for each time point and genotype). With 571 DEGs, ADB-susceptible UW2 showed the most pronounced response, while ADB-susceptible UW1 yielded the lowest number with just 194 DEGs. In addition, the analysis resulted in 395 and 500 DEGs for the two ADB-tolerant FAR3 and FS36, respectively. Complete results for the four genotypes are shown in Supplementary Tables S19 to S22. As shown in the Venn diagram (Fig. 4), transcriptional responses also differed between genotypes, 484 DEGs being exclusive for UW2 (32.4%), 419 DEGs being exclusive for FS36 (28.1%), 295 DEGs exclusively found in FAR3 (19.8%), and 147 DEGs for UW1 (9.8%). Among the shared transcripts, the bigger intersections were the 37 (2.5%) DEGs common for FAR3 and UW2, and the 32 (2.1%) DEGs common for the two ADB-tolerant genotypes FAR3 and FS36. Additionally, 12 DEGs were common for the two ADB-susceptible genotypes UW1 and UW2. Interestingly, no DEGs were found to be shared among the four genotypes. Genes included in the Venn diagram intersections shown in Fig. 4 are also presented in Supplementary Table S23 together with their descriptions from BLASTp top hits. In addition, Supplementary Table S24 combines results from each genotype, together with the clustering results and the intersections from the Venn diagram (Fig. 4).

**Fig. 5:**
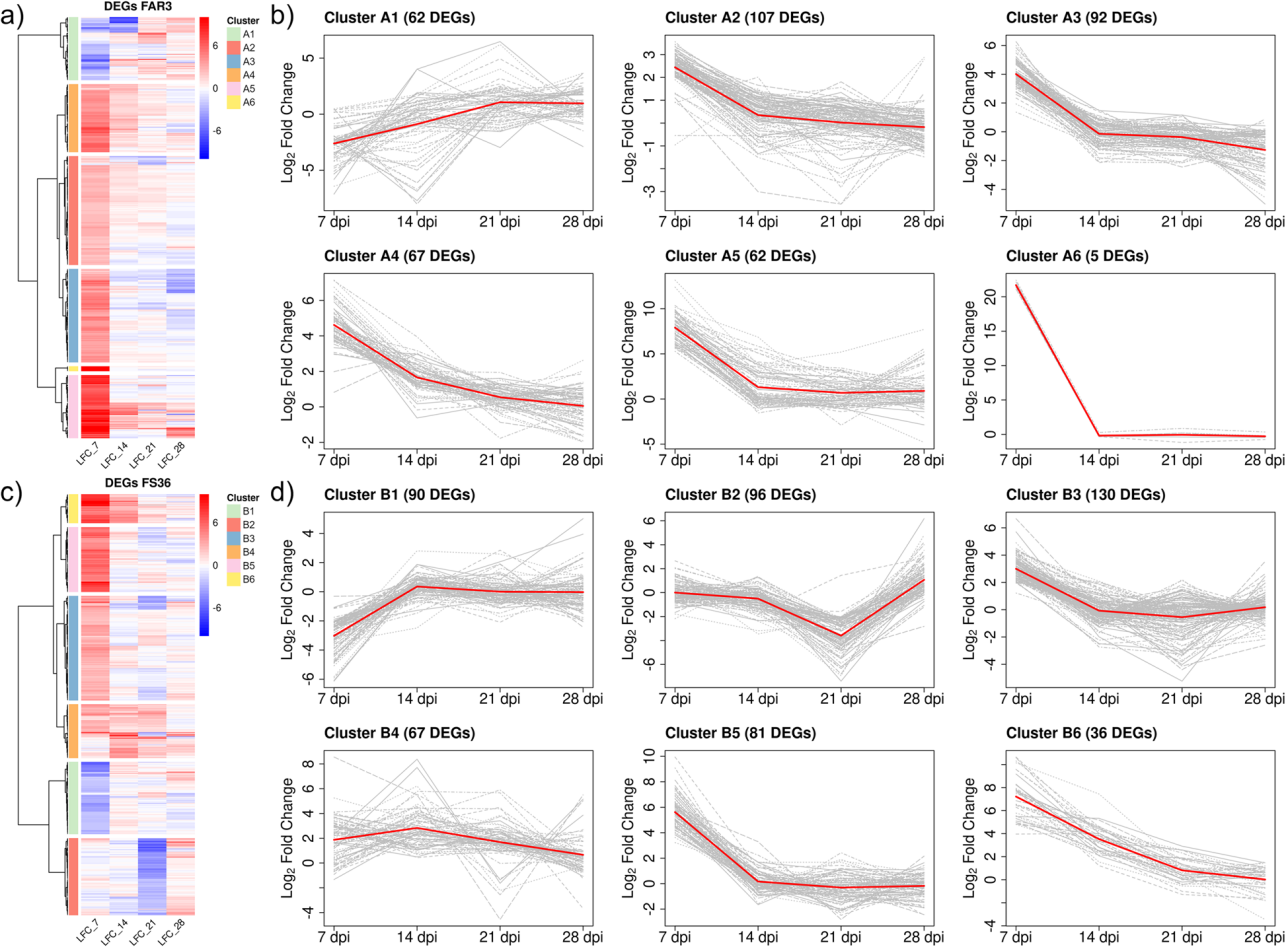
Differentially expressed genes (DEGs) in ADB-tolerant *Fraxinus excelsior* genotypes FAR3 and FS36. **a** Hierarchical clustering based on Wards minimum variance method and Euclidean distances of logarithmic fold change (LFC) values of differentially expressed genes (DEGs) obtained for ADB-tolerant genotype FAR3 after time-course gene expression analysis. LFC values were obtained for single time points (7-, 14-, 21- and 28-days post inoculation, dpi) after Wald test. **b** Linear representation of the LFC values for the six main expression profiles obtained after clustering for FAR3. **c** Hierarchical clustering based on Wards minimum variance method and Euclidean distances of logarithmic fold change (LFC) values of differentially expressed genes (DEGs) obtained for ADB-tolerant genotype FS36 after time-course gene expression analysis. LFC values were obtained for single time points (7-, 14-, 21- and 28-days post inoculation, dpi) after Wald test. **d** Linear representation of the LFC values for the six main expression profiles obtained after clustering for FS36

The temporal transcriptional profiles for each of the four genotypes were analysed by clustering the expression values (LFC) into six main clusters. In ADB-tolerant FAR3 (Fig. 5a-b), cluster A1 (62 DEGs) contained genes with an increasing trend from down-regulation in response to the infection at 7 and 14 dpi to up-regulation at 21 and 28 dpi. The remaining clusters for this genotype contained DEGs that were mostly induced at 7 dpi decreasing later at 14, 21 and 28 dpi, with clusters A2 and A3 being the largest groups with 107 and 92 DEGs, respectively. In contrast, only 5 DEGs were included in cluster A6, but showing the highest expression values (LFC > 20) at 7 dpi. In ADB-tolerant FS36, the response was found to be a bit different (Fig. 5c-d). Cluster B1 (90 DEGs) included genes that were repressed at 7 dpi, later induced from 14 to 28 dpi, while DEGs included in cluster B2 (96 DEGs) included genes showing a sharp drop in the expression at 21 dpi. Moreover, genes included in clusters B5 (81 DEGs) and B6 (36 DEGs) showed the highest LFC values at 7 dpi, although with a lower expression compared to FAR3.

**Fig. 6:**
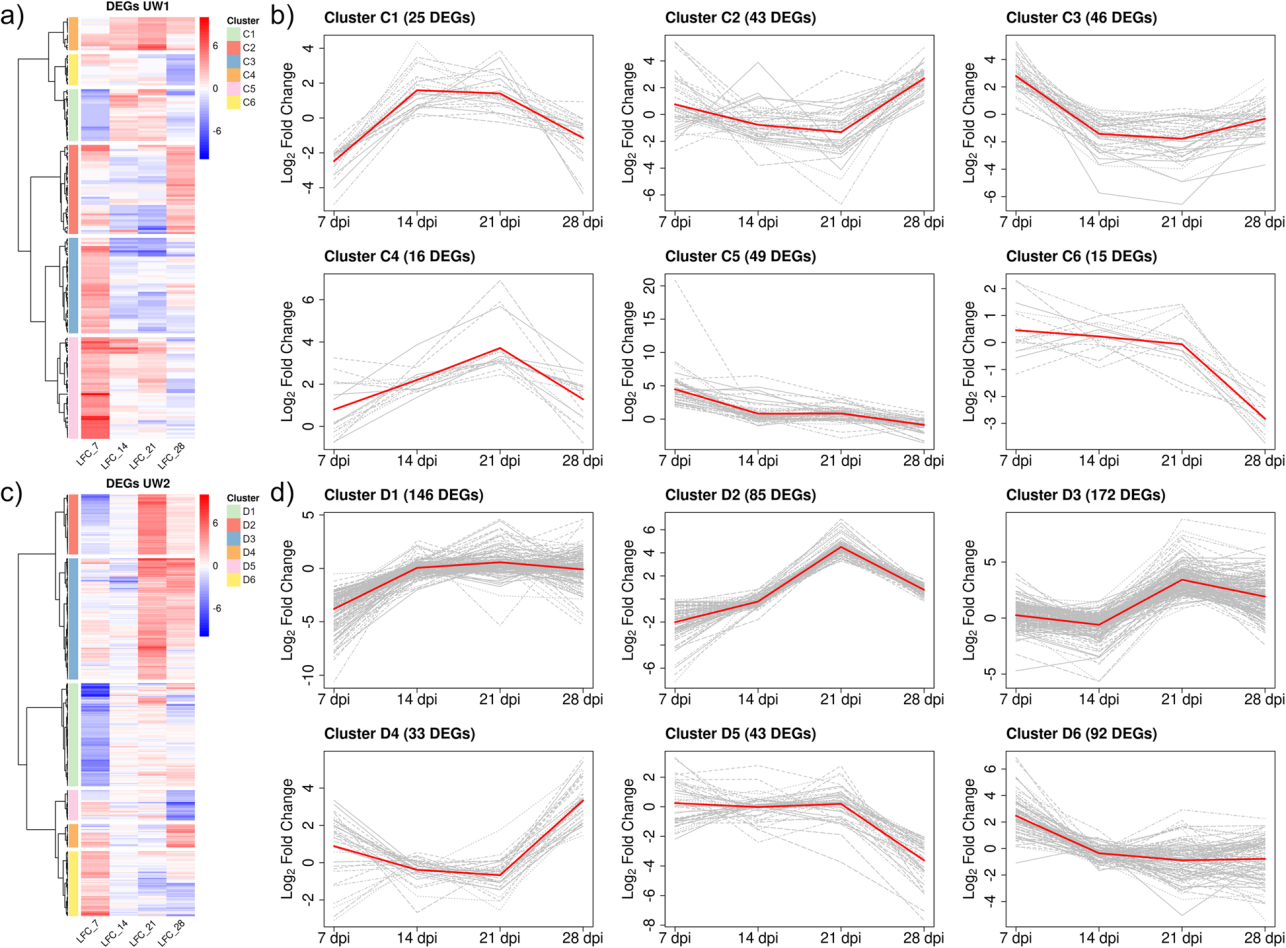
Differentially expressed genes (DEGs) in ADB-susceptible *Fraxinus excelsior* genotypes UW1 and UW2. **a** Hierarchical clustering based on Wards minimum variance method and Euclidean distances of logarithmic fold change (LFC) values of differentially expressed genes (DEGs) obtained for ADB-susceptible genotype UW1 after time-course gene expression analysis. LFC values were obtained for single time points (7-, 14-, 21- and 28-days post inoculation, dpi) after Wald test. **b** Linear representation of the LFC values for the six main expression profiles obtained after clustering for UW1. **c** Hierarchical clustering based on Wards minimum variance method and Euclidean distances of logarithmic fold change (LFC) values of differentially expressed genes (DEGs) obtained for ADB-susceptible genotype UW2 after time-course gene expression analysis. LFC values were obtained for single time points (7-, 14-, 21- and 28-days post inoculation, dpi) after Wald test. **d** Linear representation of the LFC values for the six main expression profiles obtained after clustering for UW2

**Fig. 7:**
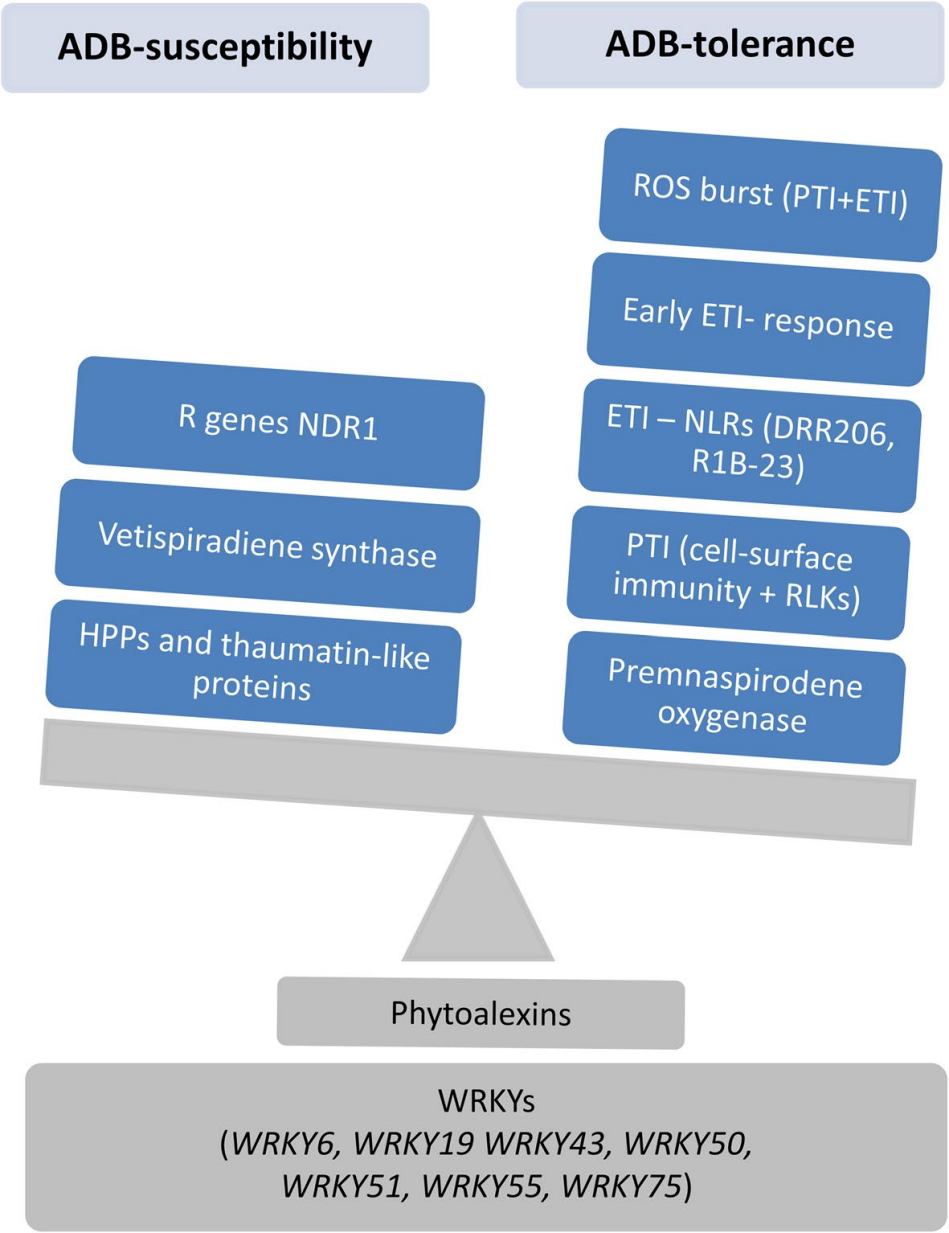
Schematic summary of the most striking transcriptomic differences between ADB-tolerant and ADB-susceptible *Fraxinus excelsior* genotypes, which were identified and discussed in the present work. All abbreviations can be found in the text

Clustering in ADB-susceptible genotypes resulted in different expression patterns. In UW1, which had the lowest number of DEGs (Fig. 6a-b), genes in cluster C1 (25 DEGs) showed repression at 7 dpi, were induced later at 14 and 21 dpi but dropped again at 28 dpi. In clusters C2 (43 DEGs) and C3 (46 DEGs), genes were mostly induced at 7 dpi, later down-regulated at 14 and 21 dpi, while genes included in cluster C5 (49 DEGs) showed the highest LFC values at 7 dpi, decreased afterwards and reached repression at 28 dpi. Compared to UW1, UW2 showed a higher proportion of repressed genes (Fig. 6c-d). Most of them were down-regulated at 7 dpi, later up-regulated, and included in clusters D1 (146 DEGs) and D2 (85 DEGs). Genes included in clusters D2 and D3 (the largest with 172 DEGs) also showed the highest LFC values at 21 dpi. Genes included in cluster D6 (92 DEGs) are also worth highlighting, as they showed an up-regulation at 7 dpi, followed by a decrease from 14 to 28 dpi. A summary of the genes involved in defensive functions is shown in Table 1 for further discussion due to their possible role in response to ADB, as well as a schematic representation of the main functionalities found in Fig. 7.

**Table 1:** Selected differentially expressed genes during the time-course response of four ash genotypes (FAR3, FS36, UW1 and UW2) to Ash Dieback. When signficant, the cluster where the gene was included is shown. Empty fields indicate no significance. Cl: cluster

GeneId	Intersect	Description from BLASTp top-hit	Abreviation	Cl. FAR3	Cl. FS36	Cl. UW1	Cl. UW2
gene30032	FAR3|FS36|UW1	elicitor-responsive protein 1-like	ERP1	A5	B6	C2	
gene32783	FAR3|FS36|UW2	berberine bridge enzyme-like 18	BBL18	A5	B6		D3
gene11790	FAR3|FS36	receptor-like serine/threonine-protein kinase SD1-8	SD1-8	A2	B3		
gene27156	FAR3|FS36	CBL-interacting serine/threonine-protein kinase 23-like	CBL23	A2	B3		
gene3603	FAR3|FS36	ethylene-responsive transcription factor ABR1-like	ABR1	A3	B3		
gene7266	FAR3|FS36	ethylene-responsive transcription factor ERF061	ERF061	A4	B6		
gene15816	FAR3|FS36	NAC domain-containing protein 2-like	NAC2	A2	B3		
gene3487	FAR3|FS36	NAC transcription factor 29-like	NAC29	A3	B4		
gene48505	FAR3|UW1	receptor-like protein kinase ANXUR1	ANXUR1	A6		C5	
gene53100	FAR3	disease resistance protein RPM1-like	RPM1	A2			
gene27923	FAR3|FS36	disease resistance response protein 206-like	RRP206	A4	B3		
gene55276	FAR3|FS36	putative late blight resistance protein homolog R1B-23 isoform X2	R1B23	A3	B5		
gene200	UW1	protein NDR1-like	NDR1			C5	
gene33030	UW2	NDR1/HIN1-like protein 10	NHL10				D4
gene33418	FS36	NDR1/HIN1-like protein 13	NHL13		B2		
gene22175	FS36	putative disease resistance RPP13-like protein 1	RPP13		B3		
gene14452	UW2	putative disease resistance RPP13-like protein 1	RPP13				D1
gene19356	FAR3	ankyrin repeat-containing protein NPR4-like	NPR4	A5			
gene6636	FS36	ankyrin repeat-containing protein NPR4-like	NPR4		B1		
gene48168	FS36	serine/threonine-protein kinase EDR1-like isoform X3	EDR1		B5		
gene34255	UW2	protein ENHANCED DISEASE RESISTANCE 2-like	EDR2				D1
gene22875	FS36	probable WRKY transcription factor 19	WRKY19		B3		
gene26550	FS36	probable WRKY transcription factor 33	WRKY33		B4		
gene24961	FAR3	probable WRKY transcription factor 43	WRKY43	A5			
gene49873	UW2	probable WRKY transcription factor 43	WRKY43				D3
gene20195	FAR3|UW1	probable WRKY transcription factor 51	WRKY51	A3		C5	
gene46629	FS36	probable WRKY transcription factor 65 isoform X1	WRKY65		B6		
gene47204	FS36|UW2	probable WRKY transcription factor 71	WRKY71		B4		D3
gene23853	FAR3	probable WRKY transcription factor 75	WRKY75	A5			
gene14628	UW1	probable WRKY transcription factor 75	WRKY75			C2	
gene46355	UW2	probable WRKY transcription factor 9	WRKY9				D3
gene31332	FS36	WRKY transcription factor 6-like	WRKY6		B5		
gene27134	UW2	WRKY transcription factor 6-like	WRKY6				D3
gene13336	FAR3	cytochrome b5	CYB-5A	A4			
gene10644	FS36	cytochrome b5, seed isoform-like isoform X2	CYB-5A		B4		
gene27492	FAR3|FS36	cytochrome P450 710A11-like	CYP710A11	A4	B3		
gene3217	UW2	cytochrome P450 71A1-like	CYP71A1				D1
gene3209	FAR3	cytochrome P450 71A3-like	CYP71A3	A4			
gene40239	FS36	cytochrome P450 76A1-like	CYP76A1		B1		
gene10406	UW1	cytochrome P450 CYP72A219-like isoform X1	CYP72A219			C2	
gene26003	FS36|UW2	cytochrome P450 CYP73A100-like	CYP73A100		B4		D3
gene14222	FAR3|UW2	vetispiradiene synthase 2-like isoform X1	HVS2	A5			D3
gene15770	FAR3	viridiflorene synthase-like	TPS31	A4			
gene11235	FAR3|FS36	premnaspirodiene oxygenase-like	HPO	A4	B4		
gene50396	UW1|UW2	thaumatin-like protein	TLP			C6	D3
gene35912	FAR3|FS36	basic endochitinase-like	CHI	A3	B5		
gene38003	UW1|UW2	ethylene-responsive transcription factor ERF087-like	ERF087			C2	D5
gene43349	FS36	MLO-like protein 2	MLO2		B4		
gene41182	UW2	MLO-like protein 4	MLO4				D1
gene8687	FS36	heavy metal-associated isoprenylated plant protein 21-like	HIPP21		B2		
gene14802	FS36	heavy metal-associated isoprenylated plant protein 22-like	HIPP22		B5		
gene23219	FS36	heavy metal-associated isoprenylated plant protein 26-like isoform X1	HIPP26		B2		
gene9908	UW1	heavy metal-associated isoprenylated plant protein 3-like isoform X1	HIPP3			C2	
gene35759	UW2	heavy metal-associated isoprenylated plant protein 4	HIPP4				D3
gene49115	UW1	heavy metal-associated isoprenylated plant protein 43-like isoform X4	HIPP43			C3	
gene50836	FS36	heavy metal-associated isoprenylated plant protein 44-like	HIPP44		B1		
gene25647	UW2	heavy metal-associated isoprenylated plant protein 45	HIPP45				D3
gene40182	FS36	heavy metal-associated isoprenylated plant protein 6-like	HIPP6		B2		
gene51200	UW2	heavy metal-associated isoprenylated plant protein 6-like	HIPP6				D6
gene7203	FS36	heavy metal-associated isoprenylated plant protein 7-like	HIPP7		B2

Each of the four sets of DEGs obtained from the four genotypes by the time-course analysis was used to perform a SEA of GO terms. In ADB-tolerant FAR3 no clear indication of GOs associated with defensive mechanisms in response to a pathogenesis process was found (Supplementary Table S25). Six GOs were overrepresented in the list of DEGs from FAR3 compared to the background, including two belonging to the biological process (BP) category, “excitatory postsynaptic potential” and “chemical synaptic transmission, postsynaptic” (both related to ionic transport through the plasma membrane in plants), and four belonging to the molecular function (MF) category, “catalytic activity”, “extracellular ATP-gated monoatomic cation channel activity”, “ATP-gated ion channel activity” and “excitatory extracellular ligand-gated monoatomic ion channel activity”. On the other hand, 22 GOs were underrepresented in DEGs, mostly belonging to the BP category, related to metabolism and biosynthesis (e.g., “cellular metabolic process”, “macromolecule biosynthetic process” and “gene expression”), and cellular component (CC) (e.g., “intracellular anatomical structure”, “intracellular organelle” and “cytoplasm”). Surprisingly, no GO terms were enriched (neither overrepresented nor underrepresented) in ADB-tolerant FS36 (Supplementary Table S26), and just two GOs were identified in ADB-susceptible UW1 (Supplementary Table S27), the overrepresented “extracellular region” and the underrepresented “intracellular anatomical structure”, both from the CC category. Finally, 11 GOs were found to be enriched in ADB-susceptible UW2 (Supplementary Table S28). Specifically, GOs “secondary metabolic process” and “catalytic activity” from BP and MF categories, respectively, were overrepresented, while the nine underrepresented GOs belonged to the CC category (e.g., “intracellular anatomical structure”, “intracellular organelle” and “cytoplasm”).

## Discussion

Differential gene expression analyses revealed that most DEGs were exclusive to specific genotypes and time points after WT (Fig. 3e). In contrast, only a few DEGs were shared among time-specific responses or between susceptible and tolerant genotypes, suggesting different transcriptional responses between ADB-tolerant and ADB-susceptible genotypes, as well as within each category. Similar results were reported in Ferrari et al. [25] with only 9 shared DEGs between the ADB-tolerant FAR3 and ADB-susceptible UW1 genotypes at 7 dpi. Moreover, [69] also found a few concordantly expressed genes between symptomatic and asymptomatic trees. The time-course analysis revealed few shared DEGs between genotypes, with no common gene found in all four genotypes. Trujillo-Moya et al. [83] observed similar results in Norway spruce in response to needle bladder rust, as well as Hernandez-Escribano et al. [38] across time points in *Pinus pinaster* infected with *Fusarium circinatum*.

In our study, we generated 4,195 Mio high-quality paired-end reads for 96 samples (average 43.70 Mio reads per sample), compared to Ferrari et al. [25] with an average of 32 Mio high-quality (after filtering) single-end reads per sample. The average mapping rate increased from 89.93% in Ferrari et al. [25] to 96.63%, probably due to a more stringent filtering of the raw sequencing data. Important differences were observed for FAR3 and UW1 genotypes. For example, the WT with adjusted *p*-value threshold set on < 0.05 (less restrictive) in Ferrari et al. [25] revealed 230 and 515 DEGs in the ADB-tolerant FAR3 and the ADB-susceptible UW1, respectively, while in this study 1043 and 518 DEGs were found at 7 dpi in FAR3 and UW1, respectively, using a more restrictive adjusted *p*-value of 0.01. These differences can be explained by the different experimental set-ups, including the use of climate chambers with controlled environmental conditions instead of the greenhouse and the use of pathogen-infected agar plugs for inoculation instead of the mycelium-covered wooden plugs. The different inoculation method with *H. fraxineus* can impact infection speed and hence the host response.

### Sparse common response among genotypes

As mentioned above, no DEGs were shared between the four genotypes, with only 20 DEGs shared among three genotypes. Most of these DEGs showed higher expression in ADB-tolerant genotypes (Fig. 7). Elicitor-responsive protein 1 (ERG1)-like (gene30032), involved in the early recognition of elicitors, was significant in FAR3, FS36 and UW1 but not in UW2 (Table 1), with higher expression at 7 dpi in the ADB-tolerant FAR3 and FS36 (clusters A5 and B6) than in ADB-susceptible UW1 (cluster C2). However, the expression pattern also differed between the two tolerant genotypes (induced at 7 and 28 dpi in FAR3, but from 7 to 21 dpi in FS36), suggesting different responses to pathogen-derived elicitors. A homologous gene was also found to be induced in tree tomato (*Solanum betaceum*) challenged by *Phytophthora betacei*. Similarly, three berberine bridge enzyme-like 18 (BBL18) genes showed similar expression in ADB-tolerant FAR3 (cluster A5, induced at 7 and 28 dpi) and FS36 (cluster B6, induced at 7 and 14 dpi), but were repressed during the early response in ADB-susceptible UW2 (cluster D3), later induced but with lower expression than in tolerant genotypes (e.g., see gene32783 in Table 1). BBL proteins, important oxidoreductases in ROS burst during PTI and ETI, are involved in pathogenesis processes in *Populus trichocarpa*, *Citrus sinensis* and *Triticum aestivum* [4],[17],[31].

Time-course analysis revealed differences between ADB-tolerant and ADB-susceptible genotypes. FAR3 and FS36 shared 32 DEGs, mostly up-regulated at 7 dpi but with higher expression in FS36 (clusters A2, A3, A4 and A5 for FAR3 and B3, B4 and B5 for FS36). These included PTI-related genes such as RLKs and Ca^2+^ influx genes (e.g., gene11790 and gene27156) (Fig. 7). Other important pathogen-responsive DEGs were involved in ethylene signaling (e.g., gene3603 and –7266), which is critical for the host response to hemibiotrophic and necrotrophic pathogens [30], and NAC transcription factors (e.g., gene15816 and –3487), with functionalities in plant immunity [90].

### Early activation of ETI based defenses in ADB-tolerant genotypes

The strong up-regulation of cell-surface immunity genes observed at 7 dpi in tolerant genotypes suggests a successful activation of PTI (Fig. 7). In FAR3, several components such as LRR-RLKs, cell wall-associated kinases (WAKs), and G-type lectin RLKs were induced at 7 dpi (clusters A2 to A5). The activation of subsequent responses, such as ROS production, extracellular Ca^2+^ influx, or MAPK activation, was also suggested by the up-regulation of specific genes at 7 dpi. In particular, the RLK ANXUR1 (ANX1) encoded by gene48505 was identified in ADB-tolerant FAR3 and ADB-susceptible UW1 but not in FS36 or UW2. In FAR3, ANX1 showed a LFC = 21.11 at 7 dpi (cluster A6), later down-regulated to a basal level (LFC ≈ 0), while it showed a lower up-regulation at 7 dpi in UW1 (LFC = 5.87; cluster C5). ANX1 is a malectin-like domain-containing receptor-like kinase that interacts with both PRR-mediated PTI and NLR-mediated ETI [48], whose crosstalk has been previously described [81],[90] suggesting an earlier ETI-related response in the ADB-tolerant genotype (Fig. 7). In *Arabidopsis*, ANX1 negatively regulates RESISTANCE TO P. SYRINGAE PV MACULICOLA1 (RPM1), an R (resistance) gene that recognizes *Pseudomonas syringae* effectors and initiates ETI and the associated hypersensitive response (HR) and programmed cell death (PCD; [27, 28]. RPM1 was found to be slightly induced as DEG at 7 dpi (LFC = 2.65) only in FAR3 (cluster A2), followed by a basal expression (LFC ≈ 0), and was also induced in an elm genotype (*Ulmus americana*) resistant to Dutch elm disease [42].

The HR, supported by the ROS burst during PTI, is a form of PCD that deprives pathogens of nutrients and limits their spread [5]. However, to prevent self-damage from autoimmunity (in the form of apoptosis and autophagy), the HR should also be limited to a local response. Among the 32 DEGs shared by FAR3 and FS36, genes involved in ETI were induced at 7 dpi. The earlier ETI activation in tolerant genotypes is supported by NLRs encoding genes, such as disease resistance response protein 206-like (DRR206; Fig. 7). One transcript (gene27923) was differentially expressed in both ADB-tolerant genotypes (clusters A4 and B3 for FAR3 and FS36, respectively) but not in ADB-susceptible genotypes. DRR206 is involved in phytoalexin production, which is crucial for plant immunity [62]. Similarly, a gene coding for late blight resistance protein R1B-23 (gene55276) was induced at 7 dpi in ADB-tolerant FAR3 and FS36 (clusters A3 and B5, respectively) but not in UW1 and UW2. This gene was induced in tree tomato (*S. betaceum*) during *P. betacei* infection [6].

Based on the N-terminal domain, NLRs are classified into two main groups, the Toll/Interleukin 1 Receptor-type (TNLs) and the Coiled-coil type (CNLs), which follow different signaling pathways [43]. Disease resistance based on TNLs requires ENHANCED DISEASE SUSCEPTIBILITY1 (EDS1), whereas immunity mediated by CNLs involves NON-RACE-SPECIFIC DISEASE RESISTANCE1 (NDR1). Interestingly, NDR1 was repressed at 21 dpi in ADB-tolerant FS36 (gene33418, cluster B2), whereas it was induced at 7 and 28 dpi in ADB-susceptible UW1 (gene200, cluster C5) and UW2 (gene33030, cluster D4), respectively (Fig. 7). Additionally, two paralogues of a putative disease resistance RPP (RECOGNITION OF PERONOSPORA PARASITICA)13-like 1 (RPP13) protein, proposed to confer downy mildew resistance in *Arabidopsis* [9], were found to be induced at 7 dpi in FS36 (gene22175, cluster B3) and repressed at 28 dpi in UW2 (gene14452, cluster D1).

### Genes involved in phytohormone-signaling and transcriptional reprogramming

Phytohormones play an important role in plant immunity. The jasmonic acid (JA) and ethylene pathways are involved in defense against necrotrophic pathogens, whereas salicylic acid (SA) is essential for defense against biotrophs and hemibiotrophs [30]. *H. fraxineus* is a hemibiotrophic pathogen that exhibits a necrotrophic behaviour during the infection based on the symptoms of affected trees [33]. The expression of JA-related genes was found to be higher in tolerant than in susceptible ash trees in [69], but both signaling pathways may be crucial during the response to ADB, especially considering the lack of a common response. Furthermore, SA is essential for both local and systemic defense and is detected by the NONEXPRESSER OF PR GENES (NPR) receptor family [20]. We found two homologues of NPR1-LIKE PROTEIN 4 (NPR4) in the ADB-tolerant genotypes, one induced at 7 dpi in FAR3 (gene19356, cluster A5) and the other repressed at the same time point in FS36 (gene6636, cluster B1). Additionally, the expression of MAP kinase ENHANCED DISEASE RESISTANCE1 (EDR1, gene48168) was up-regulated in FS36 at 7 dpi (LFC = 5.40; cluster B5). EDR1 has been proposed to be a negative regulator of disease resistance by blocking ethylene and SA-signaling [45, 80], although EDR1 mutants in *Arabidopsis* showed increased resistance to biotrophs [84] but susceptibility to hemibiotrophs and necrotrophs [39]. Thus, our results suggest that ADB-tolerance in FAR3 may primarily rely on SA-signaling, consistent with previous results [25], while JA-related genes were found in FS36, including up-regulation of EDR1, at least in the early response (7 dpi). Moreover, the ENHANCED DISEASE RESISTANCE2 (EDR2) protein is involved in controlling cell death during HR via SA-signaling [85], and two EDR2 genes were highly repressed at 7 dpi in ADB-susceptible UW2 (e.g., gene34255, LFC = -7.60).

Pathogen infection also induced WRKY transcription factors in the four genotypes, which are involved in oxidative stress and regulation of plant defenses [66]. Ferrari et al. [25] observed significant up-regulation of WRKY43, WRKY50, and WRKY75 in FAR3 and UW1. In the current work, several members of the WRKY gene family were identified as DEGs in different genotypes. For example, WRKY43 (gene24961), WRKY50 (gene1271), WRKY51 (gene20195), WRKY55 (gene17069) and WRKY75 (gene23853) were induced at 7 dpi in FAR3, and most of them were also repressed at 28 dpi (clusters A3 and A5). In UW1, WRKY51 was also induced at 7 dpi and repressed at 28 dpi (cluster C5), while WRKY75 (gene14628) was induced from 14 to 28 dpi (cluster C2). In addition, WRKY43 (gene49873) was induced at 21 dpi in UW2 (cluster D3). WRKY43 has been implicated in Ca^2+^ trafficking during pathogen infection in *Arabidopsis* [63]. WRKY50, together with WRKY51 and WRKY55, have been implicated in the activation of SA-dependent pathogenesis-related gene 1 (PR1) [27, 28, 41, 86]. Notwithstanding, WRKY75 has been suggested to participate in the JA-cascade against necrotrophic pathogens [14], suggesting a common signaling step in the ADB infection regardless of genotype-specific tolerance. In FS36, WRKY6 and WRKY19 (gene31332 and -22,875) were up-regulated at 7 dpi (clusters B3 and B5, respectively). The former is involved in early steps of infection-related responses [68], while the latter is involved in basal immunity against root-knot nematodes in *Arabidopsis* together with a TIR NLR [87]. WRKY6 and WRKY9 were induced in UW2 at 21 and 28 dpi (gene27134 and -46355, cluster D3) and showed a delayed response. WRKY9 is involved in the JA-mediated response to root rot in *Panax notoginseng* [92]. Additionally, WRKY71 (gene47204), suggested to be involved in ethylene-mediated but not specifically pathogenesis-related responses [89], was induced at 14 dpi in FS36 (cluster B4) but showed delayed up-regulation at 21 dpi in UW2 (cluster D3). WRKY65 and WRKY33 (gene46629 and -26,550), implicated in SA-mediated resistance in *Arabidopsis* [40, 50], were found to be induced in FS36 (clusters B4 and B6, respectively).

Other notable genes were those involved in producing plant secondary metabolites with a role in defense and potential tolerance to ADB, as suggested by [69]. In this study, genes involved in phytoalexin biosynthesis, such as phenylpropanoids and terpenoids, were mainly induced at early infection stages (7 and 14 dpi). In FAR3, most of these genes were found in clusters A4 and A5, coding for cytochrome b5 (gene13336), cytochrome P450 (e.g., gene27498 and -3209), viridiflorene synthase (gene15770), and vetispiradiene synthase (gene14222). Some of these genes have also been implicated in Turkey berry (*Solanum torvum*) responses to root-knot nematode infection [70]. However, the expression of these terpene biosynthesis-related genes in FS36, such as gene10644, -27,492, -40,239 or -26,003, followed different trends, with some members of the cytochrome P450 superfamily being repressed at 7 dpi (cluster B1) and at 21 dpi (cluster B2), and others induced at 7 dpi (clusters B3, B4 and B5), and from 14 to 28 dpi (also cluster B4). No viridiflorene or vetispiradiene synthases were found in this genotype. In UW1, a few terpene-related DEGs were identified (e.g., three cytochrome P450-like proteins like gene10406 in Table 1), mainly repressed from 7 to 21 dpi and later up-regulated at 28 dpi (cluster C2). In UW2, these genes were also repressed at 7 dpi, with some induced at 21 dpi (e.g., gene3217 in cluster D1 or gene26003 in cluster D3) or directly induced at this time point with basal expression at the remaining times (cluster D2).

Interestingly, a vetispiradiene synthase was highly induced at 21 and 28 dpi in UW2 (gene14222 in cluster D3 and also suggested by the enriched GO “secondary metabolic process”, Supplementary Table S28), indicating a delayed expression when compared to FAR3 which may be linked to susceptibility (Fig. 7). Up-regulation of this gene has also been described during synthesis of sesquiterpenoid phytoalexins via the mevalonate pathway in potato late blight [88]. Another notable gene related to terpene biosynthesis encodes for premnaspirodiene oxygenase, a cytochrome P450 involved in production of the antifungal phytoalexin solavetivone with detoxification activity [79],[11]. Several paralogues of this gene were found in all four genotypes, but showed an earlier up-regulation (7 and 14 dpi) in the ADB-tolerant genotypes. Of particular interest was the premnaspirodene oxygenase (gene11235), which was found to be significant among the 32 shared DEGs between FAR3 and FS36 (clusters A4 and B4, respectively), but not present in UW1 and UW2 (Fig. 7).

### Genes related to ADB-susceptibility

Although no clear correlation was found for the expression profiles between UW1 and UW2, the 12 DEGs shared between them could provide insight into susceptibility. For example, a thaumatin-like protein (gene50396) was repressed in UW1 at 28 dpi (cluster C6) but induced in UW2 at 21 and 28 dpi (cluster D3; Fig. 7). Thaumatin-like proteins are pathogenesis-related (PR) proteins involved in stress tolerance and belong to the PR5 subgroup [2]. Another PR gene was a basic endochitinase (gene35192) found in both ADB-tolerant genotypes. Additionally, the ethylene-response factor EF087-like (gene38003) was repressed at 21 dpi in UW1 (cluster C2, later induced at 28 dpi) and at 28 dpi in UW2 (cluster D5, previously induced at 21 dpi). Susceptibility can also be enhanced by host factors targeted by the pathogen to suppress resistance and facilitate the infection. These include MILDEW RESISTANCE LOCUS O (MLO), a susceptibility (S) gene associated with powdery mildew susceptibility, as loss-of-function mutants show enhanced resistance [44, 51],[72]). MLO-like protein 2 (gene43349) was found to be induced at 7 dpi in FS36 (cluster B4), while an MLO-like protein 4 (gene41182) was found to be repressed at the same time point in UW2 (cluster D1), demonstrating their potential role not only in susceptibility but in tolerance.

Heavy metal-associated plant proteins (HPPs), including those with C-terminal isoprenylation motif (HIPPs), are also linked to susceptibility [19]. In this work, several DEGs encoding HIPPs were found in ADB-tolerant FS36 (but not in FAR3) and ADB-susceptible UW1 and UW2. In FS36, HIPP6-, HIPP7- and HIPP21-like (gene40182, -7203 and –8687, all in cluster B2) were repressed at 21 dpi (cluster B2), while HIPP44-like was repressed at 7 dpi (gene50836, cluster B1), and HIPP26-like was repressed at 14 and 21 dpi (gene23219, cluster B2). HIPP22-like was induced at 7 dpi (gene14802, cluster B5). In UW1, HIPP3-like was repressed at 14 and 21 dpi (gene9908, cluster C2), while HIPP43-like was induced at 7 dpi and slightly repressed later (gene49115, cluster C3). Finally, HIPP4- and HIPP45-like were induced at 14 and 21 dpi in UW2 (gene35759 and –25,647, both in cluster D3), while HIPP6-like was induced at 7 dpi (gene51200, cluster D6). Overall, a higher expression was detected in UW1 and UW2 genotypes, especially in the latter, suggesting a possible role in ADB-susceptibility (Fig. 7).

### Benefits of transcriptomic studies on forest health

Over the last decade, transcriptomic analyses have been successfully applied for the study of important tree diseases affecting angiosperms (e.g., [24, 37, 42, 57]) and gymnosperms (e.g., [38, 47, 83, 91]). Transcriptomics has already been applied for the analysis of the molecular response of European ash trees to ADB. In [69], candidate genes involved in phytohormone signaling and secondary metabolite synthesis pathways were identified using gene expression analyses in bark tissue. In addition, associative transcriptomics were used in combination with crown damage scores to predict phenotypes based on gene expression variants [36]. In this work, we had a deeper look into DEGs related with plant immunity, while providing a valuable resource of transcriptomic data for downstream analyses such as further gene expression analyses or targeted GWAS based on candidate genes. These potential genes are useful genetic resources for the development of genetic markers to identify and breed ash genotypes with tolerance to ADB [59]. Moreover, these genetic markers could be useful for the assessment of evolutionary dynamics and to define conservation units [35, 61].

## Conclusions

In this study, we have focused on a time-course gene expression analysis during the local response to ADB in four ash genotypes with differences in susceptibility. The analysis revealed different gene expression profiles between tolerant and susceptible genotypes, as well as within these groups. On the one hand, tolerant genotypes showed an early expression of genes involved in both PTI and ETI, while susceptible genotypes exhibited a delayed response. Notably, most DEGs were exclusively identified in individual genotypes, indicating diverse strategies in the tolerance to ADB. However, shared functionalities rather than specific shared gene ids can help to disclose important pathways involved in the production of plant secondary metabolites (i.e., phytoalexins, terpenes, phenolic compounds, etc.), and these genes deserve further attention.

This research provides valuable insights into the molecular mechanisms underlying ADB tolerance in European ash trees. The identification of genes involved in phytoalexin production and other secondary metabolites with roles in plant defense, particularly in tolerant genotypes, contributes to a better understanding of the genetic basis of ADB resistance. These findings pave the way for future studies aimed at developing more effective strategies for managing ADB and breeding tolerant ash trees. These results highlight the need for further research into the genetic basis of tolerance, which could be used to design more accurate breeding programs, ultimately supporting sustainable forestry practices.

## Supplementary Information

Supplementary Material 1.

Supplementary Material 2.

Supplementary Material 3.

## Data Availability

Raw sequencing data (FASTQ) have been deposited to the National Center for Biotechnology Information (NCBI) under the BioProject accession no. PRJNA1158680, and the Sequence Read Archive (SRA) accession no. from SRR30642551 to SRR30642646 (http://www.ncbi.nlm.nih.gov/sra/). RNA-Seq counts were uploaded to the Gene Expression Ommnibus (GEO) database under the accession no. GSE278637 (https://www.ncbi.nlm.nih.gov/geo). The code and scripts used for the bioinformatic workflow of this work are available at https://github.com/vchano/fraxgen_time.course.
