# Supplementary material for: Transcriptional time-course analysis during ash dieback infection revealed different responses in tolerant and susceptible Fraxinus excelsior genotypes
Source: BMC Plant Biol. 2025 Jan 25;25:107. doi: 10.1186/s12870-025-06074-z (PMC11762065; doi:10.1186/s12870-025-06074-z)

*BMC Plant Biology –* Supplementary Material.

**Transcriptional time-course analysis during ash dieback infection revealed different responses in tolerant and susceptible *Fraxinus excelsior* genotypes**

Authors: Víctor Chano, Renata Callegari Ferrari, Tania Dominguez-Flores, Karuna Shrestha, Barbara Fussi, Hannes Seidel, Oliver Gailing, Katharina B. Budde

The following Supporting Information is available for this article:

- **Figure S1.** Percentage of ramet mortality for the four European ash (*Fraxinus excelsior*) genotypes used for the present work: ash dieback (ADB)-tolerant FAR3 and FS36 and ADB-susceptible UW1 and UW2.
- **Figure S2.** Overview of experimental preparation for inoculating *Fraxinus excelsior* trees with *Hymenocyphus fraxineus* mycelia. a) Clones growing in climate chambers. b) Superficial wounds were cut in the petioles and round agar plugs were inserted in the flap. c) A petiole mock-inoculated with sterile agar. d) A petiole inoculated with infected medium plug containing the virulent *H. fraxineus* strain. e) After 7, 14, 21, and 28 days post-inoculation, petioles were sampled.
- **Table S1** (in file Chano.etal.2024_pce_supplementary_tables_1.xlsx). Differentially expressed genes from Wald test (WT), including the number of induced and repressed genes.
- **Table S2** (in file Chano.etal.2024_pce_supplementary_tables_1.xlsx). Differentially expressed genes (DEGs) from Wald test (WT) for ash genotype FAR3, at 7 days post inoculation (dpi).
- **Table S3** (in file Chano.etal.2024_pce_supplementary_tables_1.xlsx). Differentially expressed genes (DEGs) from Wald test (WT) for ash genotype FAR3, at 14 days post inoculation (dpi).
- **Table S4** (in file Chano.etal.2024_pce_supplementary_tables_1.xlsx). Differentially expressed genes (DEGs) from Wald test (WT) for ash genotype FAR3, at 21 days post inoculation (dpi).
- **Table S5** (in file Chano.etal.2024_pce_supplementary_tables_1.xlsx). Differentially expressed genes (DEGs) from Wald test (WT) for ash genotype FAR3, at 28 days post inoculation (dpi).
- **Table S6** (in file Chano.etal.2024_pce_supplementary_tables_1.xlsx). Differentially expressed genes (DEGs) from Wald test (WT) for ash genotype FS36, at 7 days post inoculation (dpi).
- **Table S7** (in file Chano.etal.2024_pce_supplementary_tables_1.xlsx). Differentially expressed genes (DEGs) from Wald test (WT) for ash genotype FS36, at 14 days post inoculation (dpi).
- **Table S8** (in file Chano.etal.2024_pce_supplementary_tables_1.xlsx). Differentially expressed genes (DEGs) from Wald test (WT) for ash genotype FS36, at 21 days post inoculation (dpi).
- **Table S9** (in file Chano.etal.2024_pce_supplementary_tables_1.xlsx). Differentially expressed genes (DEGs) from Wald test (WT) for ash genotype FS36, at 28 days post inoculation (dpi).
- **Table S10** (in file Chano.etal.2024_pce_supplementary_tables_1.xlsx). Differentially expressed genes (DEGs) from Wald test (WT) for ash genotype UW1, at 7 days post inoculation (dpi).
- **Table S11** (in file Chano.etal.2024_pce_supplementary_tables_1.xlsx). Differentially expressed genes (DEGs) from Wald test (WT) for ash genotype UW1, at 14 days post inoculation (dpi).
- **Table S12** (in file Chano.etal.2024_pce_supplementary_tables_1.xlsx). Differentially expressed genes (DEGs) from Wald test (WT) for ash genotype UW1, at 21 days post inoculation (dpi).
- **Table S13** (in file Chano.etal.2024_pce_supplementary_tables_1.xlsx). Differentially expressed genes (DEGs) from Wald test (WT) for ash genotype UW1, at 28 days post inoculation (dpi).
- **Table S14** (in file Chano.etal.2024_pce_supplementary_tables_1.xlsx). Differentially expressed genes (DEGs) from Wald test (WT) for ash genotype UW2, at 7 days post inoculation (dpi).
- **Table S15** (in file Chano.etal.2024_pce_supplementary_tables_1.xlsx). Differentially expressed genes (DEGs) from Wald test (WT) for ash genotype UW2, at 14 days post inoculation (dpi).
- **Table S16** (in file Chano.etal.2024_pce_supplementary_tables_1.xlsx). Differentially expressed genes (DEGs) from Wald test (WT) for ash genotype UW2, at 21 days post inoculation (dpi).
- **Table S17** (in file Chano.etal.2024_pce_supplementary_tables_1.xlsx). Differentially expressed genes (DEGs) from Wald test (WT) for ash genotype UW2, at 28 days post inoculation (dpi).
- **Table S18** (in file Chano.etal.2024_pce_supplementary_tables_1.xlsx). Differentially expressed genes included in the intersections of upset plot from time-specific analysis by Wald test for the four ash genotypes, ADB-tolerant FAR3 and FS36, and ADB-susceptible UW1 and UW2. ADB: ash dieback.
- **Table S19** (in file Chano.etal.2024_pce_supplementary_tables_2.xlsx). Differentially expressed genes along the response (time-course) to infection with *Hymenoscyphus fraxineus* from likelihood ratio test performed in the ash genotype FAR3, tolerant to ADB. padj: p-value adjusted by False Discovery Rate (FDR); LFC: logarithmic fold change; ADB: ash dieback.
- **Table S20** (in file Chano.etal.2024_pce_supplementary_tables_2.xlsx). Differentially expressed genes along the response (time-course) to infection with *Hymenoscyphus fraxineus* from likelihood ratio test performed in the ash genotype FS36, tolerant to ADB. padj: p-value adjusted by False Discovery Rate (FDR); LFC: logarithmic fold change; ADB: ash dieback.
- **Table S21** (in file Chano.etal.2024_pce_supplementary_tables_2.xlsx). Differentially expressed genes along the response (time-course) to infection with *Hymenoscyphus fraxineus* from likelihood ratio test performed in the ash genotype UW1, susceptible to ADB. padj: p-value adjusted by False Discovery Rate (FDR); LFC: logarithmic fold change; ADB: ash dieback.
- **Table S22** (in file Chano.etal.2024_pce_supplementary_tables_2.xlsx). Differentially expressed genes along the response (time-course) to infection with *Hymenoscyphus fraxineus* from likelihood ratio test performed in the ash genotype UW2, susceptible to ADB. padj: p-value adjusted by False Discovery Rate (FDR); LFC: logarithmic fold change; ADB: ash dieback.
- **Table S23** (in file Chano.etal.2024_pce_supplementary_tables_2.xlsx). Differentially expressed genes included in the intersections of venn diagram from time-course analysis for four ash genotypes, ADB-tolerant FAR3 and FS36, and ADB-susceptible UW1 and UW2. ADB: ash dieback.
- **Table S24** (in file Chano.etal.2024_pce_supplementary_tables_2.xlsx). Combined table of differentially expressed genes along the response (time-course) to infection with *Hymenoscyphus fraxineus* from likelihood ratio tests performed in the four ash genotypes, ADB-tolerant FAR3 and FS36, and ADB-susceptible UW1 and UW2, including the number of clusters and the intersections from venn diagram. ADB: ash dieback; dpi: days post inoculation.
- **Table S25** (in file Chano.etal.2024_pce_supplementary_tables_2.xlsx). Enriched GO terms for ADB-tolerant genotype FAR3.
- **Table S26** (in file Chano.etal.2024_pce_supplementary_tables_2.xlsx). No enriched GO terms were found for ADB-tolerant genotype FS36.
- **Table S27** (in file Chano.etal.2024_pce_supplementary_tables_2.xlsx). Enriched GO terms for ADB-susceptible genotype UW1.
- **Table S28** (in file Chano.etal.2024_pce_supplementary_tables_2.xlsx). Enriched GO terms for ADB-susceptible genotype UW.

**Figure S1.** Percentage of ramet mortality for the four European ash (*Fraxinus excelsior*) genotypes used for the present work: ash dieback (ADB)-tolerant FAR3 and FS36 and ADB-susceptible UW1 and UW2.


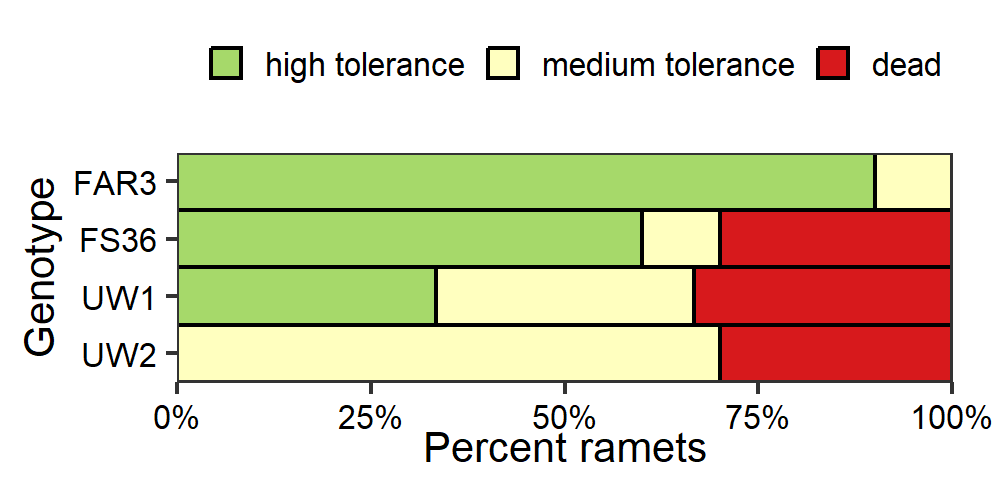


**Figure S2.** Overview of experimental preparation for inoculating *Fraxinus excelsior* trees with *Hymenocyphus fraxineus* mycelia. a) Clones growing in climate change conditions. b) Wounds were performed in the petioles and round agar plugs were inserted in the flap. c) A petiole mock-inoculated with sterile agar. d) A petiole inoculated with infected medium containing the virulent *H. fraxineus* strain. e) After 7, 14, 21, and 28 days post-inoculation, petioles were sampled.


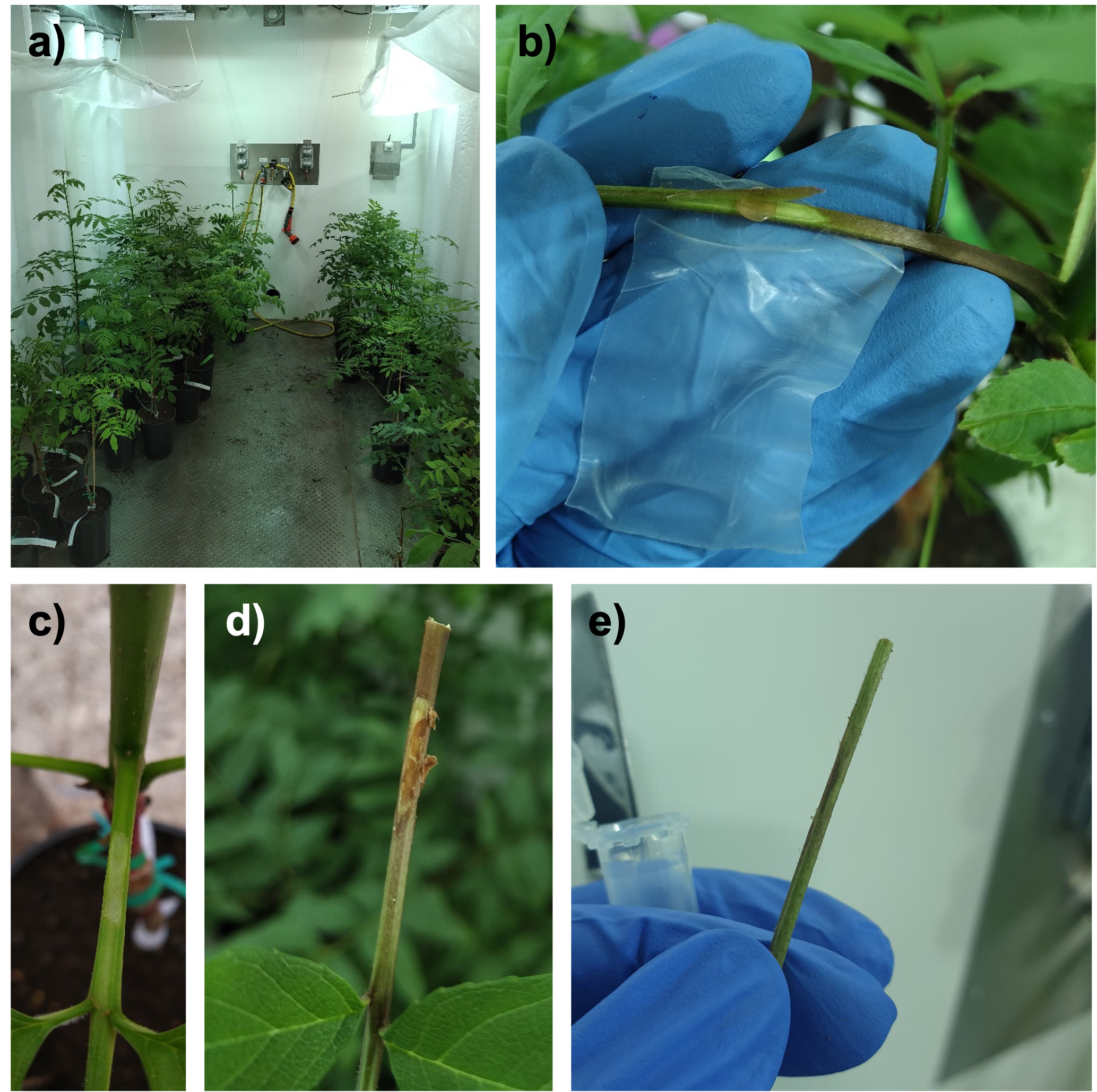

Supplement: Supplementary file 1 — Supplementary Material 1. [file 12870_2025_6074_MOESM1_ESM.docx]
